# A Non-invasive Radiomic Method Using ^18^F-FDG PET Predicts Isocitrate Dehydrogenase Genotype and Prognosis in Patients With Glioma

**DOI:** 10.3389/fonc.2019.01183

**Published:** 2019-11-14

**Authors:** Longfei Li, Wei Mu, Yaning Wang, Zhenyu Liu, Zehua Liu, Yu Wang, Wenbin Ma, Ziren Kong, Shuo Wang, Xuezhi Zhou, Wei Wei, Xin Cheng, Yusong Lin, Jie Tian

**Affiliations:** ^1^Collaborative Innovation Center for Internet Healthcare, Zhengzhou University, Zhengzhou, China; ^2^CAS Key Laboratory of Molecular Imaging, Institute of Automation, Chinese Academy of Sciences, Beijing, China; ^3^Department of Neurosurgery, Peking Union Medical College Hospital, Chinese Academy of Medical Sciences and Peking Union Medical College, Beijing, China; ^4^Engineering Research Center of Molecular and Neuro Imaging of Ministry of Education, School of Life Science and Technology, Xidian University, Xi'an, China; ^5^School of Electronics and Information, Xi'an Polytechnic University, Xi'an, China; ^6^Department of Nuclear Medicine, Peking Union Medical College Hospital, Chinese Academy of Medical Sciences and Peking Union Medical College, Beijing, China; ^7^School of Software, Zhengzhou University, Zhengzhou, China; ^8^School of Artificial Intelligence, University of Chinese Academy of Sciences, Beijing, China; ^9^Beijing Advanced Innovation Center for Big Data-Based Precision Medicine, Beihang University, Beijing, China

**Keywords:** ^18^F-FDG PET, radiomics, glioma, isocitrate dehydrogenase, non-invasive prediction

## Abstract

**Purpose:** We aimed to analyze ^18^F-fluorodeoxyglucose positron emission tomography (^18^F-FDG PET) images via the radiomic method to develop a model and validate the potential value of features reflecting glioma metabolism for predicting isocitrate dehydrogenase (IDH) genotype and prognosis.

**Methods:** PET images of 127 patients were retrospectively analyzed. A series of quantitative features reflecting the metabolic heterogeneity of the tumors were extracted, and a radiomic signature was generated using the support vector machine method. A combined model that included clinical characteristics and the radiomic signature was then constructed by multivariate logistic regression to predict the IDH genotype status, and the model was evaluated and verified by receiver operating characteristic (ROC) curves and calibration curves. Finally, Kaplan-Meier curves and log-rank tests were used to analyze overall survival (OS) according to the predicted result.

**Results:** The generated radiomic signature was significantly associated with IDH genotype (*p* < 0.05) and could achieve large areas under the ROC curve of 0.911 and 0.900 on the training and validation cohorts, respectively, with the incorporation of age and type of tumor metabolism. The good agreement of the calibration curves in the validation cohort further validated the efficacy of the constructed model. Moreover, the predicted results showed a significant difference in OS between high- and low-risk groups (*p* < 0.001).

**Conclusions:** Our results indicate that the ^18^F-FDG metabolism-related features could effectively predict the IDH genotype of gliomas and stratify the OS of patients with different prognoses.

## Introduction

Glioma is a common type of primary malignant central nervous system tumor and causes significant morbidity and mortality (1), with an incidence of 4–5 per 100,000 individuals. The prognosis of patients is grim; <50% of patients with low-grade glioma have no recurrence 10 years after diagnosis (2), and the 5-year survival rate of patients with high-grade glioma is just 5% (3). Isocitrate dehydrogenase (IDH) is emphasized as a key biomarker for glioma prediction and prognosis in the 2016 update of the WHO diagnostic criteria (4), and the overall survival (OS) of patients with IDH mutants is significantly better than the OS of those with wild-type IDH (5). The IDH biomarker is also critical for accurate glioma classification (4), planning of the scope of surgical resection (6), and guiding of the chemotherapy regimen (7, 8). Thus, accurate IDH genotype prediction may have a positive impact on the individualized treatment plan of patients with glioma.

The status of IDH mutation is currently mainly detected through immunohistochemistry, PCR product sequencing, and other technologies, using surgical or biopsy tumor samples. However, there is still an unmet clinical need for easily accessible biomarkers that can be used to acquire the underlying tumor genotype and achieve patient survival stratification accurately. Several exploratory studies have tried to use detection and analysis techniques that use circulating tumor cells, circulating tumor DNA, and serum/cerebrospinal fluid biomarkers to identify IDH mutants. Nevertheless, these studies are still at a relatively early stage (9, 10). At the same time, other studies have attempted to predict the status of IDH genotypes in patients with glioma through magnetic resonance (MR) or positron emission tomography (PET) imaging parameters, including the apparent diffusion coefficient and relative cerebral-blood-volume for MR and the tumor-to-brain ratio and time-to-peak for PET (11, 12). However, the above studies of IDH genotype identification based on image parameters lacked the necessary validation data to verify the performance of the proposed methods.

An emerging radiomic method based on the combination of artificial intelligence and medical imaging has attracted wide attention due its potential value for accurate diagnosis and prognosis assessment (13). The radiomic method aims to perform non-invasive tumor analysis by extracting a suite of quantitative features from medical images (14–16). These features include a variety of gene expression types that provide a more comprehensive description of tumor characteristics, enabling researchers to obtain an effective signature to inform objective clinical decisions (17–19). Some studies have been carried out based on MR images and radiomic methods and have demonstrated the potential value of radiomic features in predicting the gene status of gliomas (20–22). It is well-known that PET imaging is functional molecular imaging that uses tracers to visualize biological processes such as uptake of glucose, consumption of amino acid analogs, cell proliferation, etc. Specifically, ^18^F-fluorodeoxyglucose positron emission tomography (^18^F-FDG PET) reflects the uptake of glucose in tumor areas and determines the spatial distribution of radioactive PET imaging agents quantitatively *in vivo*, using elevated metabolism at the molecular level to map tumorigenic activity (23). This imaging method provides additional insight beyond MR imaging (MRI) into the biology of gliomas, which facilitates the analysis of the tumor from the perspective of glucose metabolism. In glioma research, ^18^F-FDG PET is widely applied, for example for tumor grading (24), determination of tumor extent (25), surgical planning (26), differentiation of tumor progression and necrosis (27), and prognosis prediction (28). However, to the best of our knowledge, few studies have used ^18^F-FDG PET images and the radiomic method to predict IDH genotype.

Therefore, in this study, we performed a comprehensive analysis and developed a combined model based on ^18^F-FDG PET radiomic signatures and the preoperative clinical characteristics of patients for non-invasive prediction of glioma IDH genotype status. We hypothesized that this radiomic analysis may identify differences in ^18^F-FDG metabolism between tumors with different IDH genotypes and thereby help to assess patient IDH genotype and prognosis.

## Materials and Methods

### Patients

For this retrospective study, we used database records of patients who were diagnosed with primary glioma between 2010 and 2017 at the Peking Union Medical College Hospital, Beijing, China. A total of 127 consecutive cases were included in this study according to the inclusion and exclusion criteria presented in Supplementary Methods 1 and Supplementary Figure 1. The design and protocol of the study were conducted in accordance with the Declaration of Helsinki and were approved by the Ethics Committee of Peking Union Medical College Hospital, with all requirements for informed patient consent waived. These patients were randomly divided into two groups, two-thirds (*N* = 84) in the training cohort and one-third (*N* = 43) in the validation cohort.

### IDH Mutant Detection

IDH1 and IDH2 mutations were detected postoperatively in patient tumor tissue using direct sequencing, as described by Horbinski et al. (29). DNA was isolated from formalin-fixed, paraffin-embedded tumor tissue using the Simplex OUP®FFPE DNA extraction kit (TIB, China), and the quantity was assessed by spectrophotometry using a NanoDrop 2000 (Thermo Fisher, US). Polymerase chain reaction (PCR) was accomplished with IDH1 primer (IDH1-F) 5′-TGATGAGAAGAGGGTTGAG-3′, (IDH1-R) 5′-TTACTTGATCCCCATAAGCC-3′, and IDH2 primer (IDH2-F) 5′-GACCCCCGTCTGGCTGTG-3′, (IDH2-R) 5′-CAAGAGGATGGCTAGGCGAG-3′ using the DRR007 kit (Takara, Japan) and a Verity 96-Well Thermal Cycler (Thermo Fisher, US) to amplify the fragment that contains two mutation hotspots. PCR products were treated with Exonuclease I and Antarctic Phosphatase (New England Biolabs, UK) and sequenced using a Genetic Analyzers 3500 (Thermo Fisher, US).

### ^18^F-FDG PET Data Acquisition and Tumor Segmentation

^18^F-FDG was produced *in situ* using an RDS-111 Cyclotron (CTI, US). After a fast of at least 4 h, patient blood glucose level was tested and confirmed as not exceeding the normal limit (6.4 mM). A dose of 5.55 MBq (0.15 mCi) ^18^F-FDG per kg of body weight was injected intravenously under standardized conditions in a quiet, dark room with the patient's eyes closed. An ^18^F-FDG PET/CT scan was performed 40–60 min after the ^18^F-FDG injection using a Biograph 64 TruePoint TrueV PET system (Siemens Medical Solutions, Germany). The reconstruction of PET imaging used a 336 × 336 pixel matrix, corresponding to a voxel size of 1 × 1 mm with a 3 mm slice thickness.

The three-dimensional region of interest (ROI) of every tumor was manually segmented within each slice using ITK-SNAP software (http://www.itksnap.org) by two neurosurgeons with >10 years' experience in neuro-oncology and neuro-PET, respectively, who were blinded to the final pathological result. The result of each segmentation was reviewed by a senior nuclear medicine physician with over 20 years' experience in this field. If the divergence between segmentations by the two neurosurgeons was <5%, the final ROI was determined as the overlapping region of the two ROIs, and if the divergence was more than 5%, the senior nuclear medicine physician made the final decision.

### Radiomic Feature Extraction and Feature Selection

**Figure 1:**
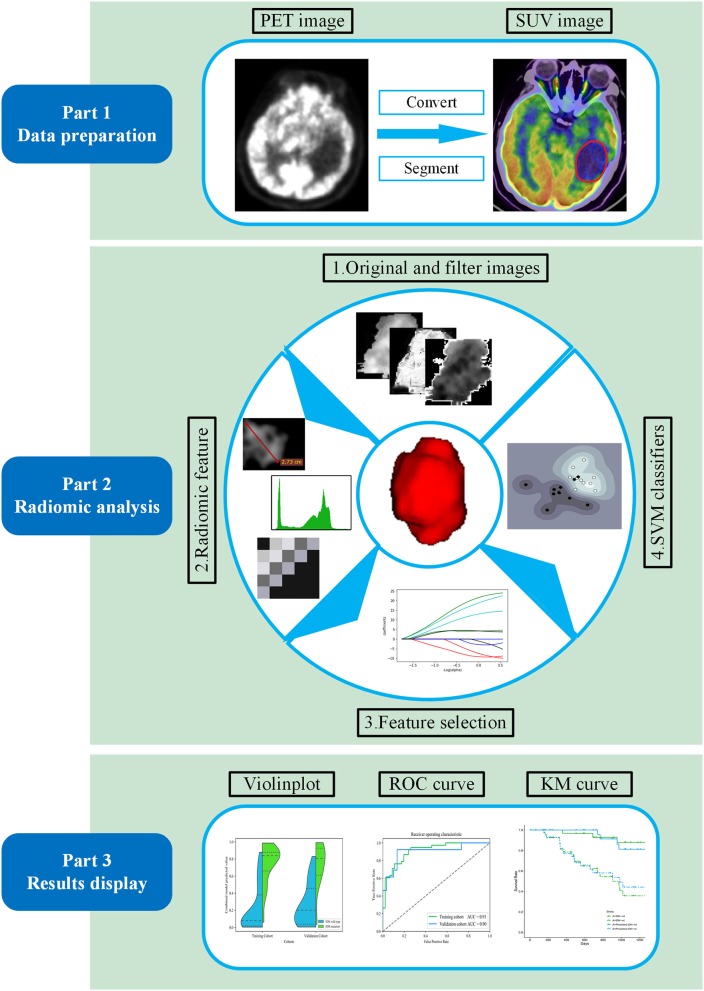
Workflow of the proposed radiomic analysis for non-invasively predicting isocitrate dehydrogenase (IDH) genotype and prognosis in glioma patients.

The radiomic analysis workflow of our study is illustrated in Figure 1. Calculations for all radiomics features were implemented from a standard uptake value (SUV) image using the open-source PyRadiomics package (https://github.com/Radiomics/pyradiomics) in Python (30). The PET image normalization method is detailed in Supplementary Method 2. Ninety-nine quantitative radiomics features were calculated from the ROI within each original SUV image, comprising 13 shape and size features, 18 first-order statistical features, and 68 texture features (22 gray-level co-occurrence matrix, 14 gray-level dependence matrix, 16-gray level run length matrix, and 16 gray-level size zone matrix features) (31). By applying eight different decomposition level wavelet filters, 688 first-order statistical and texture radiomics features were obtained. A total of 774 first-order statistical and texture features were calculated after applying the “logarithm, square, exponential, gradient, squareroot, lbp” filter. Filter descriptions and mathematical definitions for the computed radiomics features are described at (http://pyradiomics.readthedocs.io/en/latest/features.html). After applying different filters, the same number of features was extracted, including 18 first-order statistical features and 68 texture features (22 gray-level co-occurrence matrix, 14 gray-level dependence matrix, 16 gray-level run length matrix, and 16 gray-level size zone matrix features).

In order to facilitate the construction of the radiomic signature and control the feature coefficients, all radiomic feature values were normalized to between 0 and 1 according to the maximum and minimum value for the subsequent analysis. The method of feature selection with the elastic net is considered as an extension of the least absolute shrinkage and selection operator, which is appropriate in situations where the number of predictors exceeds the number of cases. With the elastic net method, which is considered an extension of the least absolute shrinkage and selection operator (32, 33) and is appropriate in situations where the number of predictors exceeds the number of cases (34). In this study, the key IDH-associated radiomics features were selected first, and then the final feature set was determined according to the greatest area under curve (AUC) value of 10-fold cross-validation. The *p*-value, based on univariate analysis, was used to assess the potential impact of clinical characteristics (Supplementary Table 1) on IDH genotype prediction.

### Model Construction and Validation

With these selected key radiomic features, a support vector machine model with a radial basis function kernel was then used to construct a radiomic signature for IDH genotype prediction in the training cohort with 10-fold cross-validation. Details of the model are provided in Supplementary Method 3. Training cohort data and the radiomic signature generated together with selected clinical features were used to establish a multiple logistic regression model for predicting the patient's IDH genotype.

The accuracy of the IDH status predictions using the above methods was assessed using the receiver operating characteristic (ROC) curve and the AUC values in the training cohort and a completely independent validation cohort. The most valuable IDH genotype prediction model was determined by comparing the predicted performance indicator values and the ROC curves (Delong's test) of the three models in the training cohort and the validation cohort and was evaluated based on the calibration curve and Hosmer-Lemeshow test (35). Decision curve analysis was used to manifest the clinical usefulness of the model by quantifying the net benefit at different threshold probabilities (36).

### Survival Analysis

Furthermore, the patients in the training and validation cohorts were divided into high- and low-risk groups according to the predicted result of the optimal model developed. The Kaplan-Meier curve was used to stratify the survival trend between patients in the two risk groups. The log-rank test was then used to verify whether there were statistical differences in survival between the two groups.

### Statistical Analysis

The differences between features were assessed using Pearson's Chi-Square tests or Fisher's exact tests for categorical variables and Student's *t*-tests or Mann-Whitney *U*-tests for continuous variables, as appropriate. The above statistical analyses were performed with SPSS Statistics software, version 18.0 (Chicago, IL, USA) or R software, version 3.4.1 (www.R-project.org). The two-tailed threshold of *p* < 0.05 was considered statistically significant.

## Results

### Clinical Characteristics

All patients underwent surgery to remove tumors, and their IDH genotype status was assessed. Of the 84 patients in the training cohort, 38 were identified as having an IDH mutant and 46 as having the IDH wild-type gene. Of the 43 patients in the independent validation cohort, 13 had an IDH mutant and 30 had the IDH wild-type gene. The baseline characteristics of the training and validation cohorts are shown in Table 1 and showed no significant differences between the two groups (*p* = 0.09–0.92), which justified their applicability as training and validation cohorts. The baseline information of patients with different IDH phenotypes is shown in Supplementary Table 2.

**Table 1:** Clinical characteristics of patients in the training cohort and validation cohort.

**Characteristics**	**Training cohort (*****n*** **=** **84)**		**Validation cohort (*****n*** **=** **43)**		***p***
	**IDH-mt (*n* = 38)**	**IDH-wt (*n* = 46)**	**IDH-mt (*n* = 13)**	**IDH-wt (*n* = 30)**	
Age (years)	43.84 ± 11.11	51.30 ± 15.33	41.85 ± 9.60	50.10 ± 20.43	0.92
Sex					0.26
Male	21	25	7	21	
Female	17	21	6	9	
Weight (kg)	67.99 ± 11.78	66.44 ± 14.72	69.00 ± 12.18	67.83 ± 13.38	0.67
Metabolism					0.42
Cystic	31	24	10	15	
Solid	7	22	3	15	
SUV_max_	10.32 ± 5.54	10.24 ± 5.12	10.09 ± 5.53	9.03 ± 4.02	0.33
SUV_mean_	4.48 ± 2.38	4.54 ± 1.86	3.80 ± 1.99	3.89 ± 1.89	0.09

*IDH, isocitrate dehydrogenase; IDH-mt, IDH mutant; IDH-wt, IDH wild-type; SUV, standard uptake value*.

*Chi-Square or Fisher's exact tests, as appropriate, were used to compare the differences in categorical variables, while independent sample t-tests or Mann-Whitney U-tests were used to compare the differences in continuous variables*.

*Age, Weight, SUV_max_„ and SUV_mean_ are represented as (mean ± standard deviation)*.

### Radiomic Feature Extraction and Feature Selection

**Figure 2:**
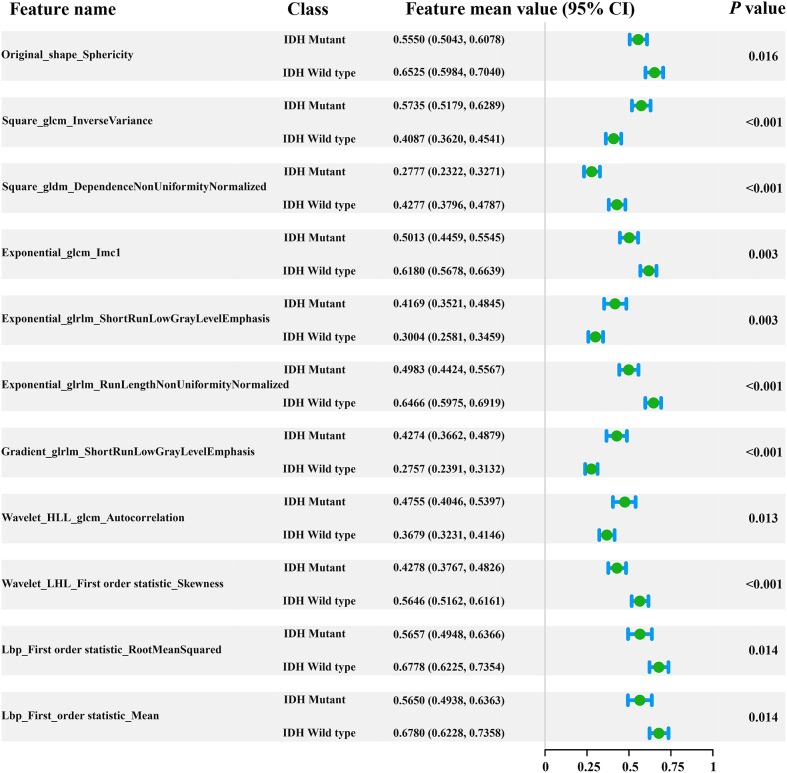
Forest plot of the 11 selected radiomic features. All features yielded significant differences in IDH mutant and IDH wild-type patients (independent *t*-test, *p* < 0.05).

In total, 1,561 radiomic features were computed in our study. After applying the elastic net method, 11 key radiomic features were selected from the training cohort for generating the radiomic signature (Figure 2), all of which showed significant differences (independent *t*-test *p* < 0.05) between IDH mutant and IDH wild-type cases (feature details are shown in Figure 2). These key radiomic features included 1 shape, 7 texture, and 3 first-order statistical features. The results of the univariate analysis of clinical characteristics revealed age and the type of tumor metabolism as significant predictors (*p* < 0.05), as presented in detail in Supplementary Table 1.

### Model Construction and Validation

**Figure 3:**
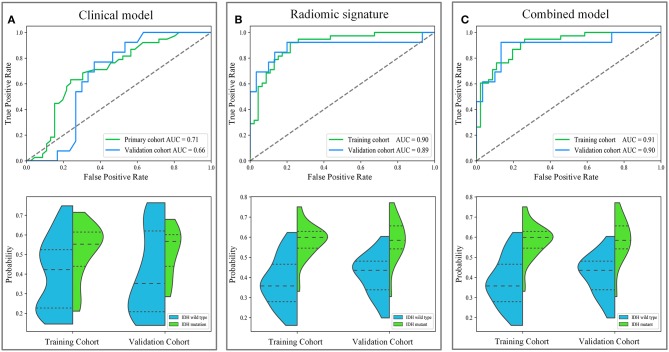
The diagnostic performance of these different models in predicting IDH genotype. The first row depicts the receiver operating characteristic (ROC) curves of the three models. The second row depicts the distribution of the IDH mutant probabilities predicted by the three models, where the horizontal dash lines are the quartiles. Subgraph **(A–C)** show the performance of clinical model, radiomic signature and combined model to predict IDH genotype, respectively.

**Figure 4:**
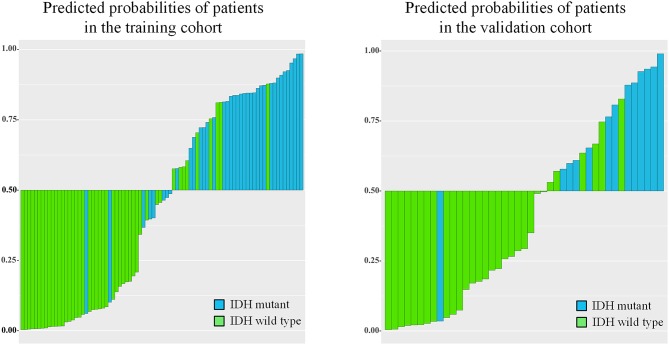
Barplots depicting the predictive performance of the combined model. A blue bar with a predicted probability value > cutoff (0.5) indicates that the model successfully identifies the IDH mutant patients; a blue bar with a predicted value < cutoff indicates that the signature fails to identify the IDH mutant patients. For the green bars, the opposite applies.

IDH genotype prediction using the above radiomic signature achieved a noteworthy result, producing AUCs of 0.904 [95% confidence interval (CI), 0.886–0.923] and 0.890 (95% CI, 0.861–0.919) in the training and validation cohorts, respectively. The predictive potential of the selected clinical characteristics was assessed by establishing and evaluating the clinical model, obtaining AUCs of 0.705 (95% CI, 0.673–0.738) and 0.664 (95% CI, 0.634–0.695) in the training and validation cohorts, respectively. A multivariable combined model was developed through the combination of age, type of tumor metabolism, and radiomic signature, which was visualized through a nomogram (Supplementary Figure 2). Detailed information such as the feature coefficients and predicted probability calculation method of the combined model are indicated in Supplementary Table 3. The combined model achieved the best result, with AUCs of 0.911 (95% CI, 0.895–0.931) for the training cohort and 0.900 (95% CI, 0.877–0.923) for the validation cohort. Figure 3 shows the ROC curves and the probability distribution of the predicted IDH mutants in the training and validation cohorts for the three models. More details on the predictive indicators obtained by these models are given in Table 2. The predictive performance of the combined model in the training and validation cohorts is also depicted by the barplots in Figure 4. Subgroup analysis shows that our model can also show good predictive performance with different glioma grades. The AUC was 0.88 and 0.93 in lower-grade tumors (WHO II and WHO III) and glioblastoma (WHO IV), respectively. Details are shown in Supplementary Figure 3.

**Table 2:** Diagnostic performance of the radiomic signature, clinical model, and combined model.

**Method**	**Cohort**	**AUC (95% CI)**	**ACC (95% CI)**	**SEN (95% CI)**	**SPE (95% CI)**
Radiomic signature	Training cohort	0.904 (0.886, 0.923)	82.1% (79.8, 84.5)	86.8.1% (83.6, 90.0)	78.3% (74.9, 81.5)
	Validation cohort	0.890 (0.871, 0.924)	81.4% (79.6, 83.9)	92.3% (89.4, 95.3)	80.0% (77.2, 82.9)
Clinical model	Training cohort	0.705 (0.673, 0.738)	66.7% (63.8, 69.5)	71.1% (66.8, 75.3)	63.0% (59.2, 67.0)
	Validation cohort	0.664 (0.631, 0.695)	65.1% (62.1, 68.0)	61.5% (56.1, 67.2)	66.7% (63.0, 70.1)
Combined model	Training cohort	0.911 (0.895, 0.931)	79.8% (77.2, 82.3)	78.9% (75.2, 82.7)	80.4% (77.0, 83.9)
	Validation cohort	0.900 (0.877, 0.923)	83.7% (81.5, 86.0)	92.3% (89.3, 95.3)	80.0% (77.1, 82.9)

*95% CI, 95% confidence interval; AUC, area under the curve; ACC, accuracy; SEN, sensitivity; SPE, specificity*.

**Figure 5:**
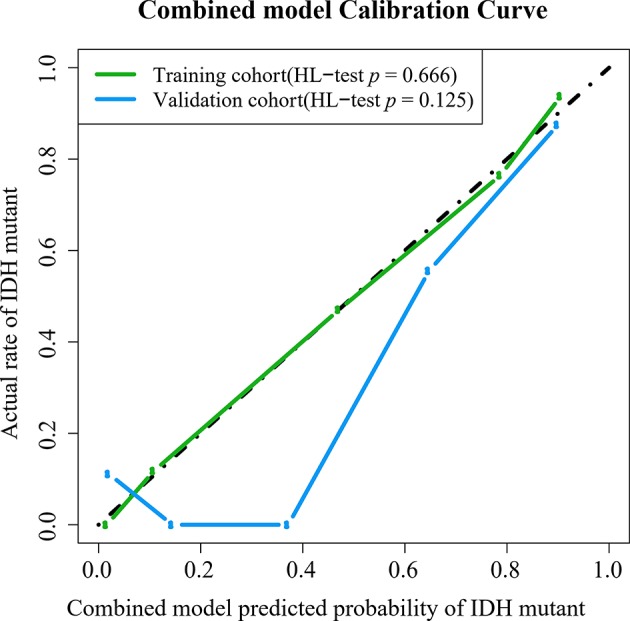
Calibration curves of the combined model in the training and validation cohorts.

Based on the results shown in Table 2 and Supplementary Table 4, the radiomic signature and the combined model showed significantly better discrimination performance (*p* < 0.05) than the clinical model alone according to the AUCs in the training and validation cohorts. Here, our results also confirm that the combined model, with more incorporated information, had the highest AUC value and showed more obvious differences in the predicted probability distribution trends of patients with different genotypes in the two cohorts. The combined model calibration curve displayed good agreement between prediction and observation in the training and validation cohorts, and the Hosmer-Lemeshow test did not show a significant difference (*p* > 0.05), demonstrating a good fit in both cohorts (Figure 5). As shown in Supplementary Figure 4, decision curves were used to demonstrate the benefits of the combined model. We found that if the threshold probability of clinical decision was >0% or >8% in training or validation cohorts, then patients would benefit more from the combined model than if genotype was not predicted.

### Survival Analysis

**Figure 6:**
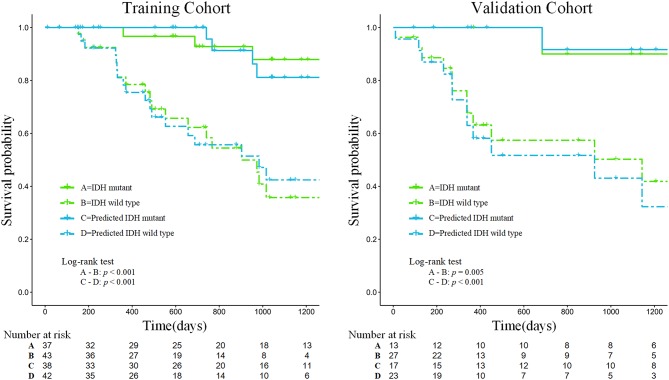
Kaplan-Meier analysis of overall survival according to the actual IDH status and the IDH status predicted by the combined model in the training and validation cohorts.

Our results suggest that the combined model not only has great potential for predicting IDH genotypes but can also help to stratify the OS of patients through Kaplan-Meier analysis (Figure 6). The predicted value of the combined model divided patients into high-risk (predicted probability <0.5) and low-risk (predicted probability ≥0.5) groups. Meanwhile, our results indicated significant statistical differences in the OS of patients, using a log-rank test between the two groups in the training and validation cohorts (*p* < 0.05).

## Discussion

In this study, we obtained 11 metabolism-related radiomic features that could reflect significant differences in different IDH genotypes of gliomas. Specifically, we developed a combined model that links the above metabolic features and clinical information to predict the IDH genotype of a glioma effectively. Moreover, our results also demonstrate that there is a significant correlation between the probabilities predicted with the combined model and patient prognosis.

**Figure 7:**
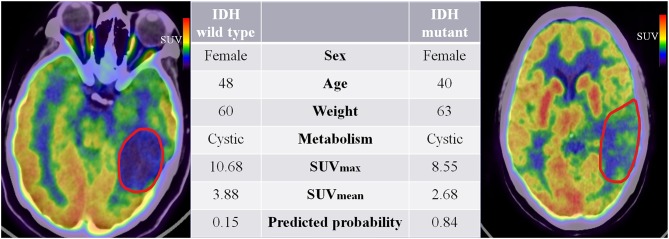
^18^F-fluorodeoxyglucose positron emission tomography images of two patients with IDH mutant and IDH wild-type status. The red boundaries in the images were manually delineated as the region of interest. All the clinical characteristics and predicted probabilities of the combined model are presented in the center table.

Our study extended previous radiomic studies on predicting IDH genotypes in patients with glioma, which predominantly linked quantitative features based on MR images to predict glioma patient IDH genotype. Yu et al. (20) showed that a radiomic study based on 110 T2-FLAIR MR images was potentially useful for non-invasive prediction of IDH genotype in grade II gliomas. Zhang et al. (21) used a combination of radiomic features based on multiparameter MRI and clinical features to predict IDH genotype in 120 high-grade gliomas. Lu et al. (22) used 214 MR images from The Cancer Image Archive and 70 collected preoperative MR images to predict the IDH mutant in low-grade gliomas. However, these studies do not fully reflect the advantages of non-invasive prediction because they all used knowledge of pathological tumor grade to select patients. The difference is that our study extracts quantitative features based on PET images that reflect tumor FDG metabolic information. Moreover, the combined model is a promising method for predicting a patient's IDH genotype and does not require prior selection of the patients based on pathological grade. Figure 7 illustrates a comparison of two representative patient cases with similar image and clinical representation; the combined model effectively distinguished between the individual with an IDH mutant and the IDH wild-type patient.

PET imaging is widely used in clinical tumor therapy and can non-invasively provide information related to tumor metabolism, predicting the progression and recurrence of glioma more effectively than MRI (37). ^18^F-FDG PET imaging can show alterations in the tumor microenvironment glucose metabolism, also known as the oncological Warburg effect (38). The differences in metabolic microenvironments between the two genotypes of glioma were reflected by the radiomic signature that combined 11 prominent high-dimensional radiomic features. The results show that the combined model has extraordinary predictive potential and can combine more information to enhance and perfect the predictive ability for IDH genotype; thus, as an indicator parameter, it may provide important predictive power for future IDH prediction. Until now, radiomic studies using ^18^F-FDG PET images for IDH genotype classification of gliomas have not been well-described in the literature. Our study clarifies the association between the radiomic features of ^18^F-FDG metabolism and IDH genotypes and has achieved noteworthy predictive and prognostic performance.

Our findings were in line with previous radiomic studies showing that features of PET images are potentially useful for solving clinical problems (39, 40). For example, the radiomic feature sphericity is a measure of how spherical tumors are and, here, shows the metabolic shape of the tumor. A recent radiomic study demonstrated that the sphericity feature based on ^18^F-FDG PET is associated with low therapeutic benefit and survival in colorectal cancer (40). Results from our study suggest that there are also significant differences in the shape feature based on ^18^F-FDG PET images in gliomas with different IDH genotypes (*p* = 0.016, Student's *t*-test). The sphericity of IDH-mutant glioma is lower than that of the IDH wild-type. These conclusions indicate that radiomic features based on ^18^F-FDG PET images play an important role, have robust applicability in solid tumor analysis, and may serve as valuable indicators to assist clinicians in making decisions.

Furthermore, the prognostic value of ^18^F-FDG PET textural features before treatment has been confirmed in several types of extracranial tumors (41, 42). It is well-known that patients with gliomas of different IDH genotypes differ in their survival times. According to follow-up information on patients, our study found that the survival curves predicted by the combined model achieved similar performance to the survival curves of patients with actual IDH genotypes, which could be used to effectively stratify the prognosis of patients (see Figure 6). Therefore, the predicted outcome of the combined model we developed was proved to be an independent risk factor for prognosis, providing a new method for predicting the prognosis of patients with glioma and showing promise as a prognostic biomarker.

Nevertheless, our study has several limitations. We used retrospective data and did not combine our analysis with baseline CT and MRI features. As this was a single-center study, larger data sets from multiple centers should be interrogated to assess the potential clinical utility of our model further. Moreover, large datasets based on multi-modal imaging may be used for refining the model to improve its predictive performance. Furthermore, although our PET-based imaging method had good predictive performance, the clinical implications of these radiomic features are currently difficult to interpret.

In summary, our results confirm that the radiomic analysis of PET images reflecting glucose metabolism in gliomas could reveal metabolic differences among gliomas with different IDH genotypes, which provides the possibility of non-invasive identification of IDH genotypes in patients. Moreover, we found a strong association between the predicted probabilities and the OS of patients, which further proved the prognostic value of the combined model.

## Data Availability Statement

The datasets of this study will not be made publicly available because they are not yet complete.

## Ethics Statement

The studies involving human participants were reviewed and approved by Peking Union Medical College Hospital. Written informed consent from the participants' legal guardian/next of kin was not required to participate in this study in accordance with the national legislation and the institutional requirements. Written informed consent was not obtained from the minor(s)' legal guardian/next of kin for the publication of any potentially identifiable images or data included in this article.

## Author Contributions

JT, YL, and XC: conception and design. YaW, YuW, WMa, ZK, and XC: collection and assembly of data. JT, YL, WMu, ZhL, LL, ZeL, XZ, SW, and WW: data analysis and interpretation. LL, JT, WMu, YaW, YL, and XC: manuscript writing.

### Conflict of Interest

The authors declare that the research was conducted in the absence of any commercial or financial relationships that could be construed as a potential conflict of interest.
