# Supplementary material for: A Non-invasive Radiomic Method Using 18F-FDG PET Predicts Isocitrate Dehydrogenase Genotype and Prognosis in Patients With Glioma
Source: Front Oncol. 2019 Nov 14;9:1183. doi: 10.3389/fonc.2019.01183 (PMC6869373; doi:10.3389/fonc.2019.01183)

**Supplementary Material**

**Supplementary Methods 1.** Inclusion and exclusion criteria for the study.

The patient inclusion criteria were as follows:

1) histopathologically diagnosed primary glioma with no previous history of central nervous system tumors;

2) availability of adequate paraffin embedded postoperative tumor tissue available for the detection of IDH1 and IDH2 mutant;

3) pre-operative PET brain scans.

Exclusion criteria were:

1) due to the effect of motion artifacts, patient’s PET images too blurred to be useable;

2) other tracer except for 18F-FDG was used for the PET image;

3) radiotherapy, chemotherapy, or other cancer treatment delivered before PET image acquired.

**Supplementary Methods 2.** The normalization formula of PET image.

SUV = $\frac{\left( PETpixel*RescaleSlope+RescaleIntercept \right)*1000*(exp(\frac{0.693}{RadionuclideHalfLife}*TimeInterval))}{RadionuclideTotalDose/PatientWeight}$

PETpixel：Pixel values in PET images.

RescaleSlope: Rescaling slope of pixel value in PET image.

RescaleIntercept: Rescaling intercept of pixel value in PET image.

TimeInterval: The interval between the injection time of contrast medium and the scanning time of image.

RadionuclideTotalDose: Injection measurement of contrast medium.

PatientWeight: The patient's weight.

**Supplementary Methods 3.** Detailed description of modeling.

For the model, the parameters of misclassification penalty C and width G were selected from [2^−10^, 2^-9^, 2^-3^, …, 2^9^,2^10^] through Grid Search with ten-fold cross-validation loops. The optimal parameters of C and G were determined by maximizing the area under the receiver operating characteristic curve (AUC). The optimal model parameters C and G were to set at 2^8^ and 2^-3^, respectively, by ten-fold cross-validation in the primary cohort.

**Tables**

**Supplementary Table 1.** Univariate logistic analysis of all the clinical characteristics.

| Clinical characteristics | Odds Ratio (95%CI) | Univariate analysis *P-*value |
| --- | --- | --- |
| Sex | 1.038 (0.438-2.461) | 0.933 |
| Age | 0.409 (0.196-0.856) | 0.018* |
| Weight | 1.143 (0.697-1.872) | 0.597 |
| Metabolism | 0.246 (0.090-0.672) | 0.006* |
| SUVmax | 1.248 (0.828-1.879) | 0.290 |
| SUVmean | 1.039 (0.926-1.165) | 0.517 |

**P* < 0.05

Abbreviations: CI, confidence interval; SUV, standard uptake value.

**Supplementary Table 2. Detailed information for patients with different IDH phenotypes.**

| **Characteristics** | **IDH mutant (N = 51)** | **IDH wild-type (N = 76)** |
| --- | --- | --- |
| Age | 43.33±10.69 | 50.82±17.40 |
| Gender |  |  |
| Male | 28 | 46 |
| Female | 23 | 30 |
| Grade |  |  |
| Ⅰ | 1 | 6 |
| Ⅱ | 28 | 10 |
| Ⅲ | 18 | 21 |
| Ⅳ | 4 | 39 |
| Median survival time  (day) | 925 | Not reach  (>1200) |
| MGMT (119) |  |  |
| Mutant | 41 | 19 |
| Wild-type | 5 | 54 |
| KI-67 (104) |  |  |
| ≥10% | 17 | 43 |
| < 10% | 28 | 16 |
| SUVmax | 17.20±46.45 | 9.456±4.124 |
| SUVmean | 8.439±30.09 | 4.331±1.993 |

Age, SUVmax, SUVmean, Prediction probability of combine model are represented as mean ± standard deviation

**Supplementary** **Table 3.** Variables, coefficients and calculation method of the combined model.

| Combined Model | | |
| --- | --- | --- |
| Intercept and Variable | β | Odds Ratio (95%CI) |
| Intercept | -7.358 |  |
| Radiomics signature | 16.397 | 52.737 (9.553-291.130) |
| Age | -0.008 | 0.584 (-1.319-0.969) |
| Metabolism | -0.464 | 0.629 (0.151-2.626) |
| Predicted probability = sigmoid (-7.358 + Radiomics signature$\times$16.397 - Age$\times$0.008 - Metabolism$\times$0.464) | | |

Note: β is the regression coefficient.

Abbreviations: CI, confidence interval.

**Supplementary** **Table 4.** The comparison of different models in the training and validation cohorts by Delong’s test.

|  | Training cohort  (*p*-value) | Validation cohort (*p*-value) |
| --- | --- | --- |
| Clinical model vs. Radiomic signature | 0.001* | 0.025* |
| Clinical model vs. Combined model | 0.0003* | 0.016* |

Note: **P* < 0.05.

**Figures**

**Supplementary Figure 1.** The inclusion and exclusion process of this study.

**
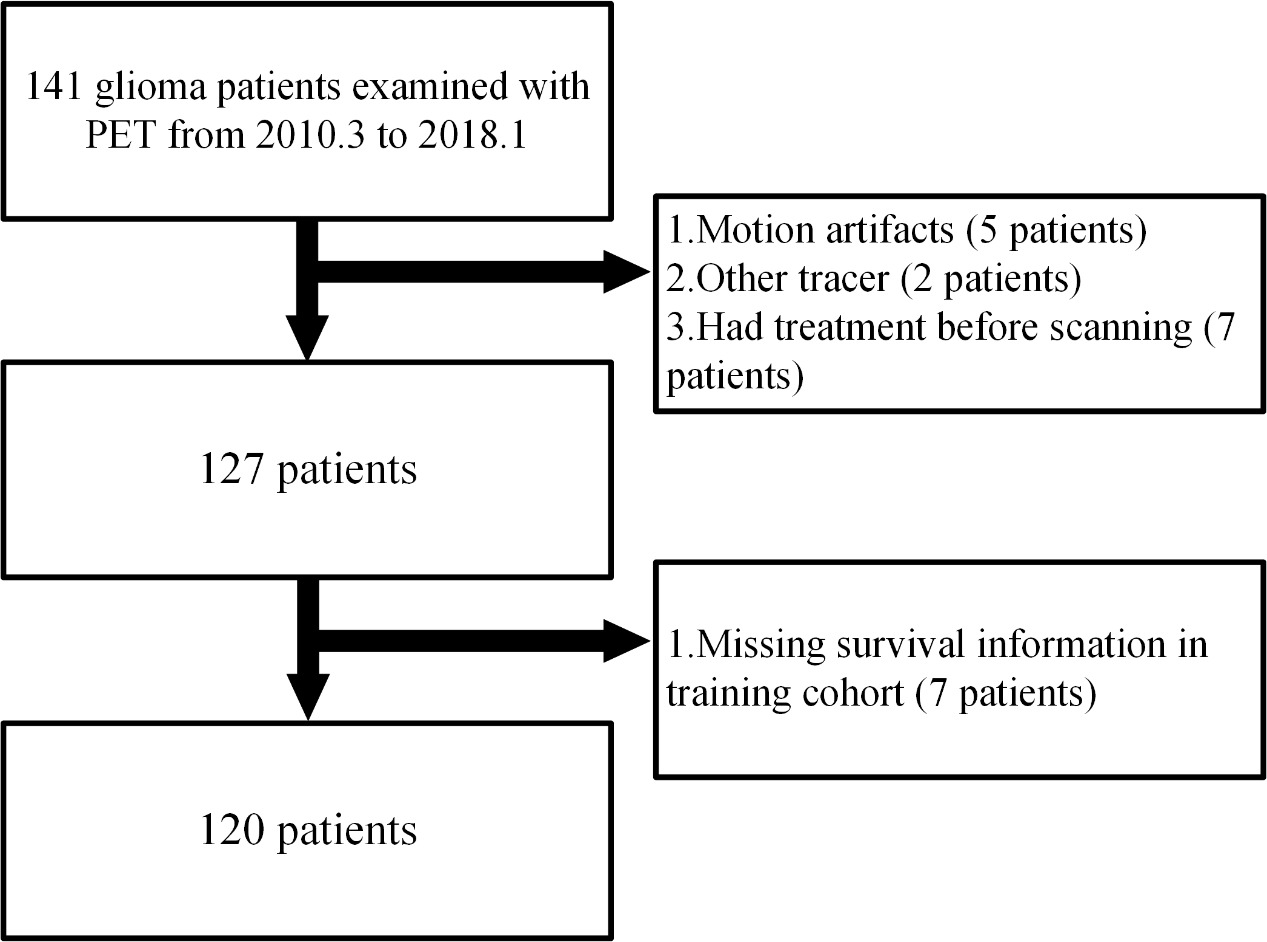
**

**Supplementary Figure 2.** The nomogram combined age, type of tumor metabolism and radiomic signature.


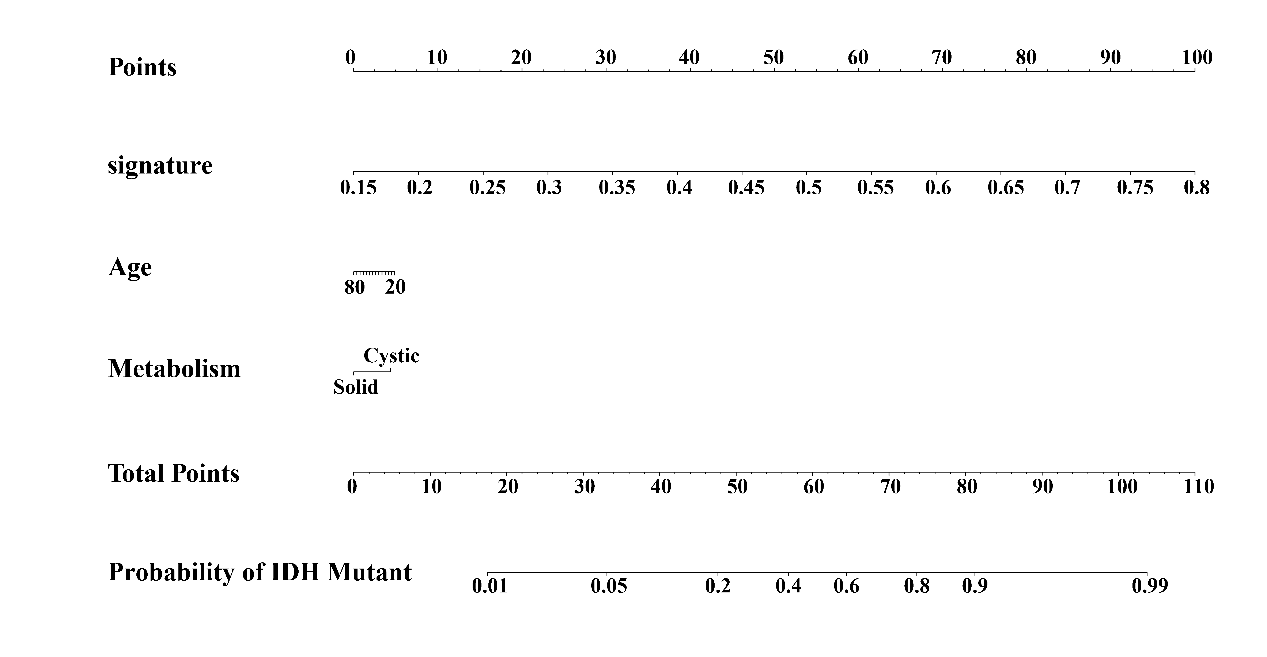


**Supplementary Figure 3.** The predictive performance of models for different grades of gliomas.
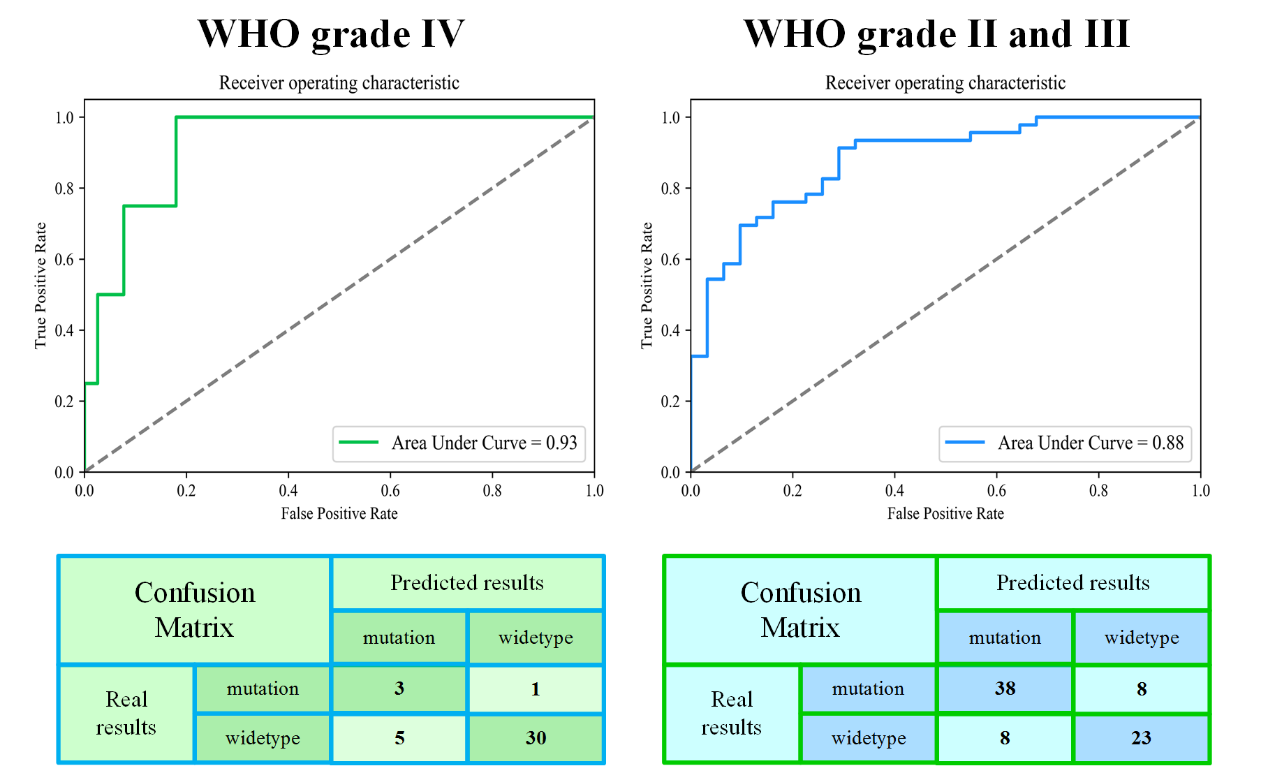


**Supplementary Figure 4.** Decision curve analysis for the combined model in the training and validation cohorts.


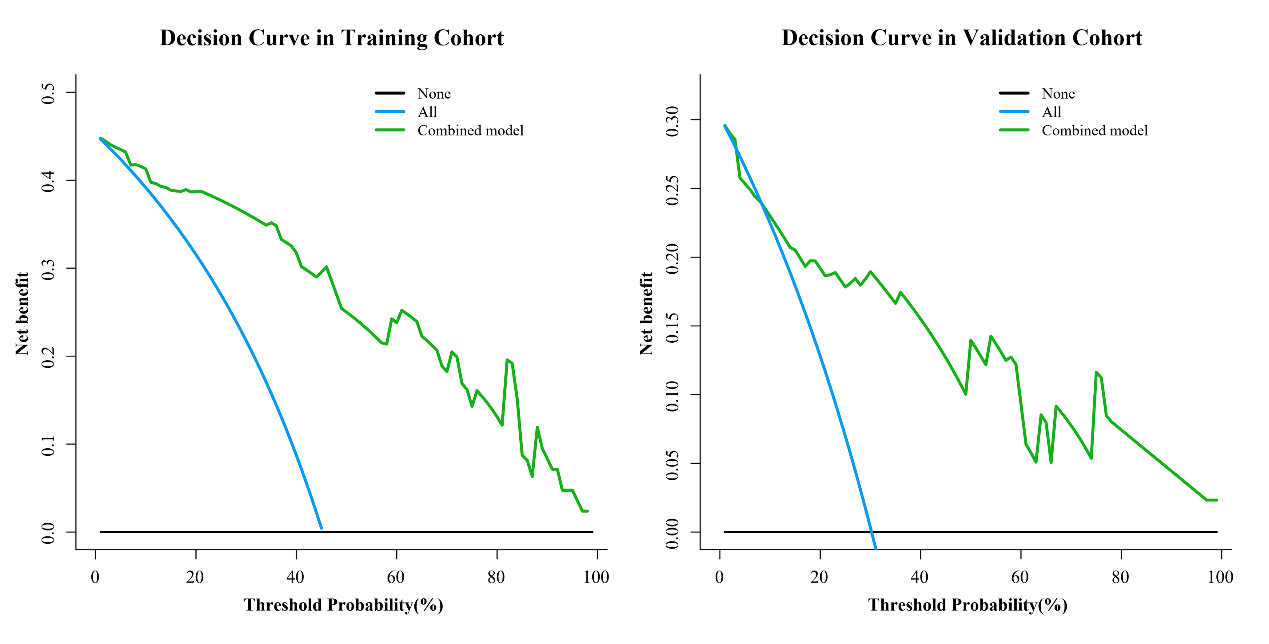

Supplement: Supplementary file 1 [file Table_1.DOCX]
